# Multi-scale image analysis and prediction of visual field defects after selective amygdalohippocampectomy

**DOI:** 10.1038/s41598-020-80751-x

**Published:** 2021-01-14

**Authors:** Bastian David, Jasmine Eberle, Daniel Delev, Jennifer Gaubatz, Conrad C. Prillwitz, Jan Wagner, Jan-Christoph Schoene-Bake, Guido Luechters, Alexander Radbruch, Bettina Wabbels, Johannes Schramm, Bernd Weber, Rainer Surges, Christian E. Elger, Theodor Rüber

**Affiliations:** 1 Department of Epileptology, University Hospital Bonn, Sigmund-Freud-Str. 25, 53127 Bonn, Germany; 2 Clinic for Neurology and Palliative Medicine, Municipal Hospital Köln-Merheim, Cologne, Germany; 3 Department of Neurosurgery, RWTH University Aachen, Aachen, Germany; 4 Department of Neurology, University of Ulm and Universitäts- and Rehabilitationskliniken Ulm, Ulm, Germany; 5 Department of Pediatric Kidney, Liver and Metabolic Diseases, Hannover Medical School, Hannover, Germany; 6 Center for Development Research, University of Bonn, Bonn, Germany; 7 Department of Neuroradiology, University Hospital Bonn, Bonn, Germany; 8 Department of Ophthalmology, University Hospital Bonn, Bonn, Germany; 9 Medical Faculty, University Hospital Bonn, Bonn, Germany; 10 Institute of Experimental Epileptology and Cognition Research, University Hospital Bonn, Bonn, Germany; 11 Department of Neurology, Epilepsy Center Frankfurt Rhine-Main, Goethe University Frankfurt, Frankfurt am Main, Germany; 12 Center for Personalized Translational Epilepsy Research (CePTER), Goethe-University Frankfurt, Frankfurt am Main, Germany

**Keywords:** Brain imaging, Magnetic resonance imaging, Neurosurgery, Epilepsy, Translational research

## Abstract

Selective amygdalohippocampectomy is an effective treatment for patients with therapy-refractory temporal lobe epilepsy but may cause visual field defect (VFD). Here, we aimed to describe tissue-specific pre- and postoperative imaging correlates of the VFD severity using whole-brain analyses from voxel- to network-level. Twenty-eight patients with temporal lobe epilepsy underwent pre- and postoperative MRI (T1-MPRAGE and Diffusion Tensor Imaging) as well as kinetic perimetry according to Goldmann standard. We probed for whole-brain gray matter (GM) and white matter (WM) correlates of VFD using voxel-based morphometry and tract-based spatial statistics, respectively. We furthermore reconstructed individual structural connectomes and conducted local and global network analyses. Two clusters in the bihemispheric middle temporal gyri indicated a postsurgical GM volume decrease with increasing VFD severity (FWE-corrected p < 0.05). A single WM cluster showed a fractional anisotropy decrease with increasing severity of VFD in the ipsilesional optic radiation (FWE-corrected p < 0.05). Furthermore, patients with (vs. without) VFD showed a higher number of postoperative local connectivity changes. Neither in the GM, WM, nor in network metrics we found preoperative correlates of VFD severity. Still, in an explorative analysis, an artificial neural network meta-classifier could predict the occurrence of VFD based on presurgical connectomes above chance level.

## Introduction

Temporal lobe epilepsy (TLE) is the most common focal epilepsy affecting 25% to 40% of all epilepsy patients^1,2^. Approximately 40% of TLE patients are pharmaco-resistant and the superiority of epilepsy surgery as compared to pharmaco-therapy has been repetitively shown^3–5^. The two most commonly applied procedures are the anterior temporal lobectomy and selective amygdalohippocampectomy (sAH)^6^. While several studies have reported no difference in patients becoming postoperatively seizure-free^6^, the theoretical advantage of sAH lies in the postoperatively lower cognitive loss due to preservation of the temporal cortex and the underlying white matter. sAH may be performed using a transsylvian^7^ or a subtemporal approach^8^. The advantage of the subtemporal as compared to the transsylvian approach is that a partial disconnection of the temporal stem can be avoided. Here, a portion of the fusiform gyrus is removed to gain access to the mesial temporal structures. Between 60 and 80% of patients are rendered seizure-free after sAH^4,5,9,10^. However, visual field deficits (VFD) have been reported to postoperatively occur in 15% to 100% of patients undergoing resective surgery in the temporal lobe^11–13^, precluding the ability to drive a car even in patients who are permanently seizure-free. These VFD, which usually manifest as contralateral homonymous upper quadrant anopia, often referred to as ‘pie in the sky’^14^, result from the spatial proximity of the Meyer's loop (ML) to the resection cavity in the temporal lobe. Patients who have undergone sAH using a subtemporal approach have been reported to show a lower rate of postoperative VFD as compared to those who have undergone sAH using a transsylvian approach^15^. With an estimated number of 100.000 TLE patients being potentially amenable to epilepsy surgery every year only in the United States^4^, an understanding of how the damage occurs and on how to prevent it during surgery is of vast clinical relevance. Beside its relevance on clinical grounds, the observation of structural changes following neurosurgery is of interest in the context of neurodegeneration and neuroplasticity^16^ and has been aspired by the authors of several studies: while it has been generally asserted, that VFDs are more likely after epilepsy surgery in the left temporal lobe due to hemispherical asymmetry of the ML^12^, various studies have employed Diffusion Tensor Imaging and tractography to delineate the ML and anatomically relate it to either anatomical landmarks or to the resection cavity^17–22^. Resection size and the Euclidean distance between the temporal pole and the most anterior part of the ML are the most promising correlates/predictors of postoperative VFD^17,22^. However, a multi-modal, data-driven approach to identify structural underpinnings of perioperative VFD in different tissue types and on multiple scales has not yet been taken. Here, we apply several whole-brain analyses to imaging and perimetry data of patients undergoing sAH to probe for presurgical gray (GM) and white matter (WM) predictors of postoperative VFD. Furthermore, we aim to describe direct as well as indirect effects of the surgical intervention on both the level of voxels as well as structural connectomes and investigate how they relate to VFD. In an explorative analysis, we further aim to preoperatively predict postoperative VFD using a combination of structural connectomics and supervised machine learning algorithms.

## Results

### Clinical group differences

Of the 28 patients included in the study, 21 showed postsurgical VFD (11 incomplete homonymous quadrantopia, 6 complete homonymous quadrantopia, 4 incomplete homonymous hemianopia) while the other 7 showed no VFD in the automated Goldmann perimetry. VFD and no VFD patient groups did not differ significantly in age, gender, duration of epilepsy or surgery-scan interval (all *p* > 0.05). Subtemporal and transsylvian surgery procedure groups did not differ in the mentioned demographic variables (*p* > 0.05). However, patients who underwent sAH using a subtemporal access showed less severe VFDs (*p* < 0.05). Postoperative resection size and the preoperative Euclidean distance between the temporal pole and the most anterior part of Meyer’s loop were tested in a regression analyses with the presence of VFD as dependent variable yielding non-significant results (both *p* > 0.45).

### VLSM results

**Figure 1:**
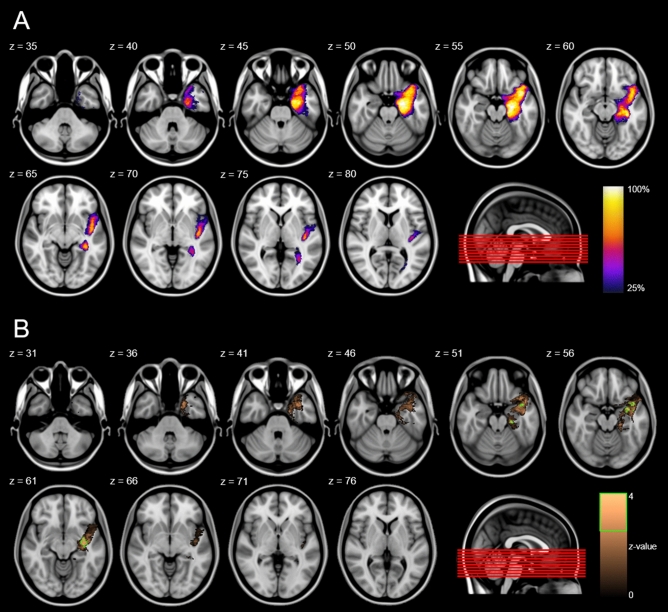
Canonical lesion mask and voxel-based lesion symptom mapping. (**A**) Canonical lesion mask after non-linear registration of all patients showing the overall lesion extent. (**B**) VLSM analysis showing a significant association between lesioned voxels and postsurgical VFDs (FWE-corr. *p* < 0.05). Green outlines indicate clusters reaching significance. z indicates axial level in MNI space.

Our VLSM analysis including all 28 manual lesion masks showed a significant association between lesioned voxels and postsurgical VFD severity in the ipsilesional external capsule and the uncinate fasciculus (FWE-corrected *p* < 0.05; Fig. 1B; volume = 423 mm^3^). Two smaller significant clusters were found in the GM of the ipsilesional temporal pole (FWE-corrected *p* < 0.05; volume = 41 mm^3^) and parahippocampal gyrus (FWE-corrected *p* < 0.05; volume = 25 mm^3^).

### VBM results

**Figure 2:**
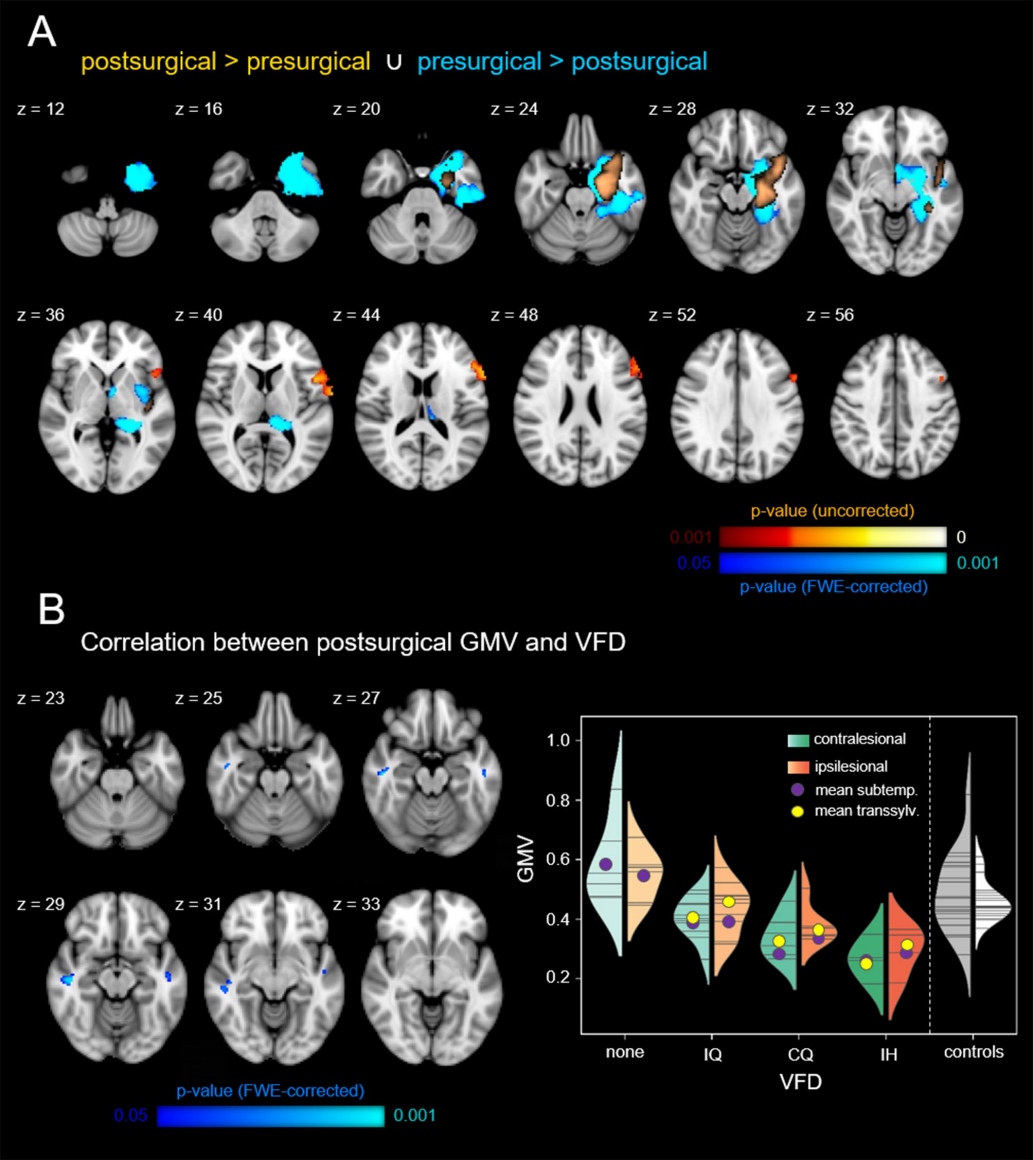
Voxel-based morphometry results. (**A**) Gray matter volume comparison of pre- and postsurgical T1-weighted scans of the transsylvian patient subgroup (FWE-corr. *p* < 0.05). (**B**) Correlation analysis between postsurgical gray matter volume and visual field deficit: the lower the gray matter volume, the higher the deficit (FWE-corr. *p* < 0.05). The canonical lesion mask is shown in bronze. Violin plots show the distributions of mean gray matter volumes within the contra- and ipsilesional cluster with each horizontal line representing the readout of one patient/control. z indicates axial level in MNI space. *GMV* gray matter volume, *IQ* incomplete homonymous quadrantopia, *CQ* complete homonymous quadrantopia, *IH* incomplete homonymous hemianopia.

Using a permutation-based paired *t*-test comparing pre- and postsurgical T1-weighted scans for the subgroup of patients who underwent a transsylvian surgical procedure (n = 18), we found a significant decrease in ipsilesional GM volume in our patient cohort (Fig. 2A). The largest cluster extended over large parts of subcortical structures, namely the ipsilesional caudate, putamen, pallidum, and thalamus. Apart from subcortical structures, the cluster of postsurgical GM decrease furthermore covered parts of the insular cortex as well as the inferior temporal and middle temporal gyrus (all FWE-corrected *p* < 0.001). The opposite contrast of a postsurgical GM volume increase resulted in a cluster covering the ipsilesional inferior frontal gyrus which, however, did not survive FWE-correction (uncorrected *p* < 0.001). Clusters remained significant after exclusion of patients with a surgery-scan-interval larger than 12 months (see Suppl. Fig. S2A).

In a second analysis, probing for a linear relationship between the degree of VFD and postsurgical GM volume, we found two significant clusters in the posterior division of both the ipsi- and the contralesional middle temporal gyrus showing a GM volume decrease with increasing VFD degree (FWE-corrected *p* < 0.05; Fig. 2B). This linear relationship can be described for both the transsylvian and subtemporal patient cohort. The opposite contrast as well as the same contrasts applied to the presurgical T1 scans did not yield any significant results.

### TBSS results

**Figure 3:**
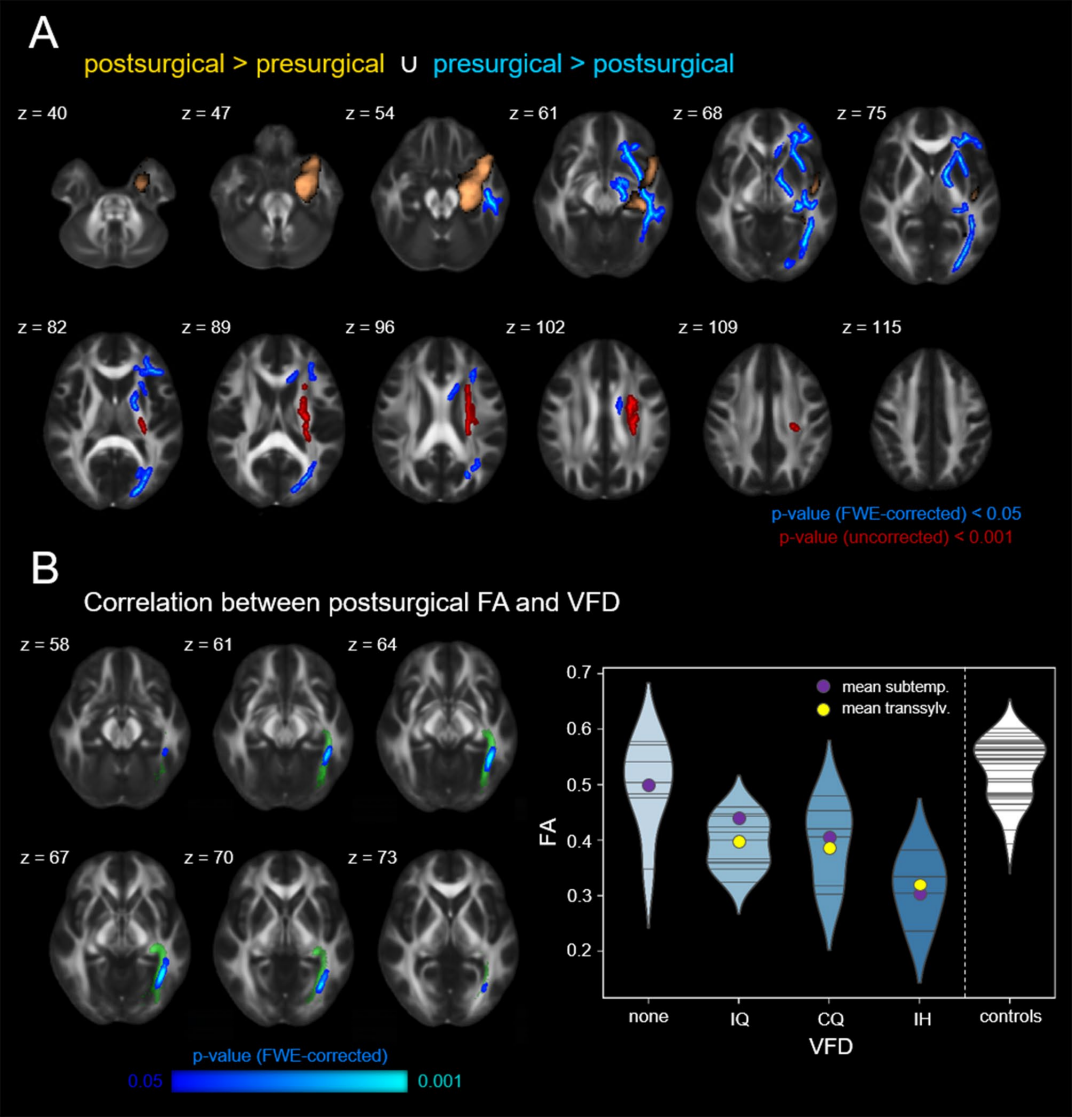
Tract-based spatial statistics results. (**A**) Comparison of fractional anisotropy in pre- and postsurgical FA maps of the transsylvian patient subgroup (FWE-corr. *p* < 0.05). (**B**) Correlation analysis between postsurgical fractional anisotropy and visual field deficit: the lower the fractional anisotropy, the larger the visual field deficit (FWE-corr. *p* < 0.05). The trajectory of the ipsilesional optic radiation as determined by probabilistic tractography is depicted in green. The canonical lesion mask is shown in bronze. Violin plots show the distributions of mean FA values within the significant cluster with each horizontal line representing the readout of one patient/control. z indicates axial level in MNI space. *FA* fractional anisotropy, *IQ* incomplete homonymous quadrantopia, *CQ* complete homonymous quadrantopia, *IH* incomplete homonymous hemianopia.

Parallel to the VBM analysis, we conducted a permutation-based paired *t*-test comparing pre- and postsurgical FA of the transsylvian subgroup. Similar to the GM changes described above, we found significantly decreased FA-values in large parts of the ipsilesional temporal and inferior frontal lobe (FWE-corrected *p* < 0.05; Fig. 3A). Clusters extended over the inferior and superior longitudinal and fronto-occipital fasciculus, as well as the anterior thalamic radiation and the uncinated fasciculus. The opposite contrast, however, yielded a significant cluster of postsurgically increased FA in the ipsilesional corona radiata, including especially the corticospinal tract. This cluster, however, did not survive FWE-correction (uncorrected *p* < 0.001). All clusters remained significant after exclusion of patients with a surgery-scan-interval larger than 12 months (see Suppl. Fig. S2B).

Testing for a linear relationship between FA and the degree of VFD, we found a single cluster showing an FA decrease with increasing extent of the VFD (FWE-corrected *p* < 0.05; Fig. 3B). The cluster coincided with the location of the sagittal stratum within the trajectory of the ipsilesional optic radiation as determined by probabilistic tractography. The linear relationship can be appreciated in both the transsylvian and subtemporal patient group. The opposite contrast as well as the correlation analysis on presurgical DTI scans did not yield any significant results.

### Connectivity differences between groups

Comparing pre- and postsurgical mean connectivity matrices, sAH can be appreciated in the zeroed postsurgical connections including amygdala and hippocampus (see Supplementary Fig. S1). Apart from this obvious observation, a slight overall drop in streamline count of connections within the ipsilesional hemisphere (upper left quadrant of connectivity matrices) in both the VFD and no VFD patient group can be seen. However, sole visualization of the connectivity matrices does not yield obvious differences between the two patient groups.

**Figure 4:**
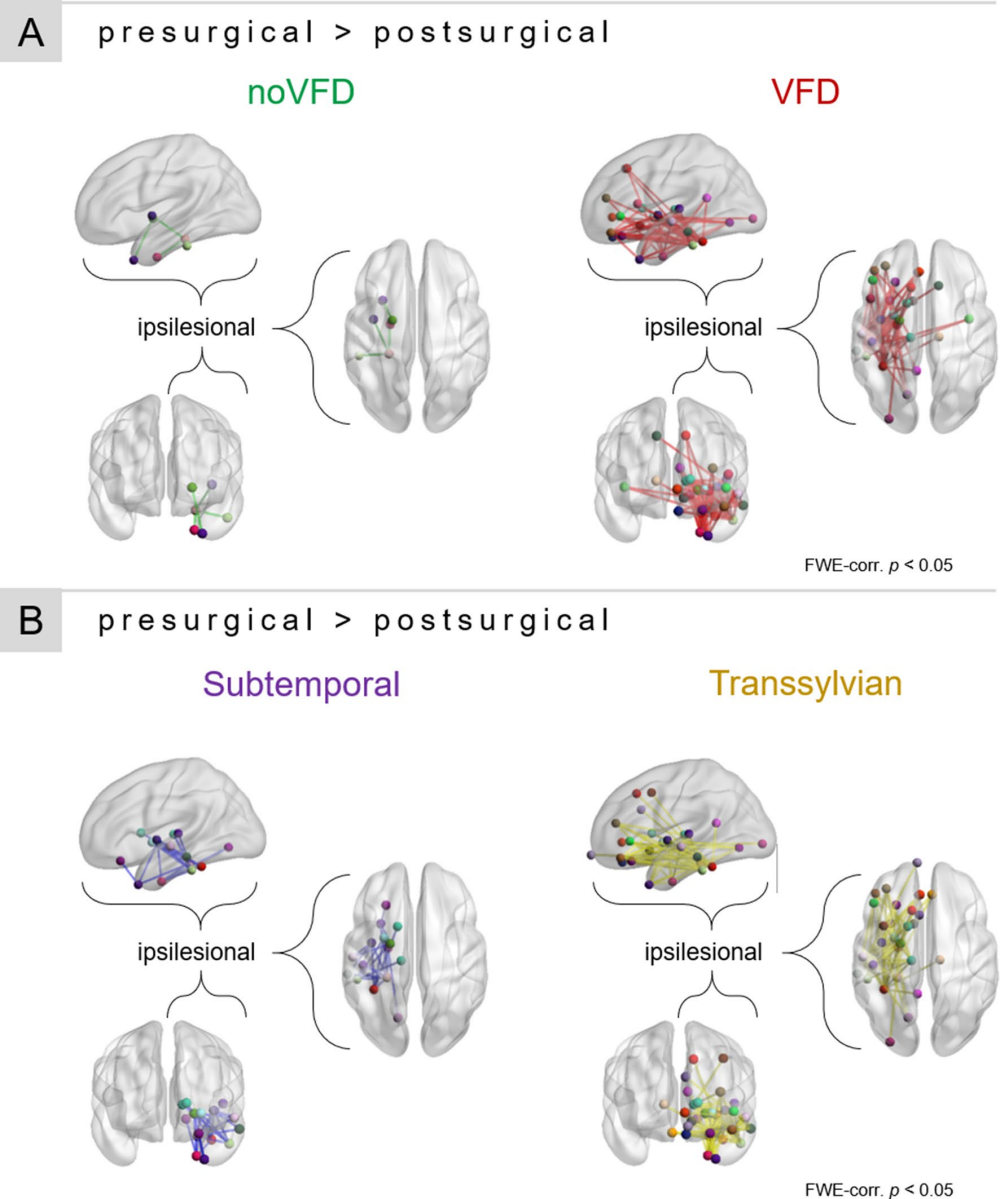
Connectivity-based statistics results. (**A**) Patients with postsurgical VFD showed a more widespread decrease in streamline count in the longitudinal comparison of pre- and postsurgical connectomes than patients without VFD. (**B**) Patients who underwent a transsylvian sAH showed more widespread connectivity decreases than patients who underwent a subtemporal approach.

Using permutation-based paired *t*-tests between pre- and postsurgical scans, a decrease in streamline count of four edges including six nodes within the ipsilesional hemisphere was found in patients showing no VFD after sAH (FWE-corr. *p* < 0.05, see Fig. 4A). In contrast to that, patients with postsurgical VFD showed an extensive loss of connectivity in a total of 73 edges involving 28 different brain regions (FWE-corr. *p* < 0.05; see Tables S1 and S2 for a list of all affected edges). Affected edges covered most of the ipsilesional temporal lobe, subcortical and prefrontal areas as well as temporo-occipital connections. Additionally, three brain regions from the contralesional hemisphere were included, namely the superior temporal gyrus, superior frontal gyrus, and pericalcarine cortex. No significantly increased streamline counts were found in the opposite contrast.

Splitting the sample according to surgical procedures, a similar pattern of connectivity differences in the pre- and postsurgical connectome comparisons can be appreciated: after subtemporal sAH, a significant decrease in streamline count was seen in 24 strictly ipsilesional edges spanning 15 nodes mostly involving the temporal lobe and subcortical brain regions (FWE-corr. *p* < 0.05, see Fig. 4B). In comparison to that, a more widespread loss in in connectivity was found after transsylvian sAH, showing decreased streamline counts in 70 mostly ipsilesional edges involving 29 brain regions, two of them on the contralesional hemisphere (fusiform gyrus, superiortemporal gyrus) (FWE-corr. *p* < 0.05, see Tables S3 and S4 for a list of all affected edges). The opposite contrast did not yield any significant results.

### Graph-theoretic differences between VFD and noVFD group

Global graph-theoretic measures were read out for a cross-sectional comparison of patients with and without postsurgical VFD in both pre- and postsurgical networks. Despite the large deviation in the longitudinal comparison, no significant differences were found between the two groups, neither in presurgical nor postsurgical networks, at a Bonferroni-corrected *p*-value threshold of 0.005. Also, a more lenient threshold of uncorrected *p* < 0.05 did not yield any significant differences.

### Classifier performances on VFD prediction

Off-the-shelf classifier performances on presurgical prediction of postsurgical VFD differed vastly: due to the unbalanced dataset, most classifiers showed a high sensitivity in positively predicting VFD ranging from 66.67 to 90.41%, only few classifiers were able to distinguish patients without postsurgical VFD above chance level, yielding specificity scores of maximally 57.14%. Gradient Tree Boosting and AdaBoost algorithms showed the strongest performance of individual classifiers, both with a total accuracy of 82.14% (sensitivity = 90.41%, specificity = 57.14%). However, none of the individual classifier reached significance in the permutation test (see Table 1 for a full performance report).

**Table 1:** Classifier performances.

Model	TP	FP	TN	FN	Specificity	Sensitivity	PPV	NPV	Accuracy	F1 score	*p*
k-nearest neighbors	17	7	0	4	0.00	80.95	70.83	0.00	60.71	0.76	0.94
Gaussian Naive Bayes	20	6	1	1	14.29	95.24	76.92	50.00	75.00	0.85	0.14
Logistic regression	16	6	1	5	14.29	76.19	72.73	16.66	60.71	0.74	0.7
Decision tree	18	4	3	3	42.86	85.71	81.82	50.00	75.00	0.84	0.21
SVM	14	6	1	7	14.29	66.66	70.00	12.50	53.57	0.68	0.86
Random forest	19	7	0	2	0.00	90.41	73.08	0.00	68.86	0.81	0.95
Extremely randomized trees	17	6	1	4	14.29	80.95	73.91	20.00	64.29	0.77	0.76
Gradient tree boosting	19	3	4	2	57.14	90.41	86.36	66.67	82.14	0.88	0.13
AdaBoost	19	3	4	2	57.14	90.41	86.36	66.67	82.14	0.88	0.13
ANN meta-classifier	20	1	6	1	**85.71**	**95.24**	**95.24**	**85.71**	**92.86**	**0.95**	*0.03

Bold values indicate best performing classifier.

*TP* = true positives, *FP* false positives, *TN* true negatives, *FN* false negatives, *PPV* positive predictive value, *NPV* negative predictive value.

**Figure 5:**
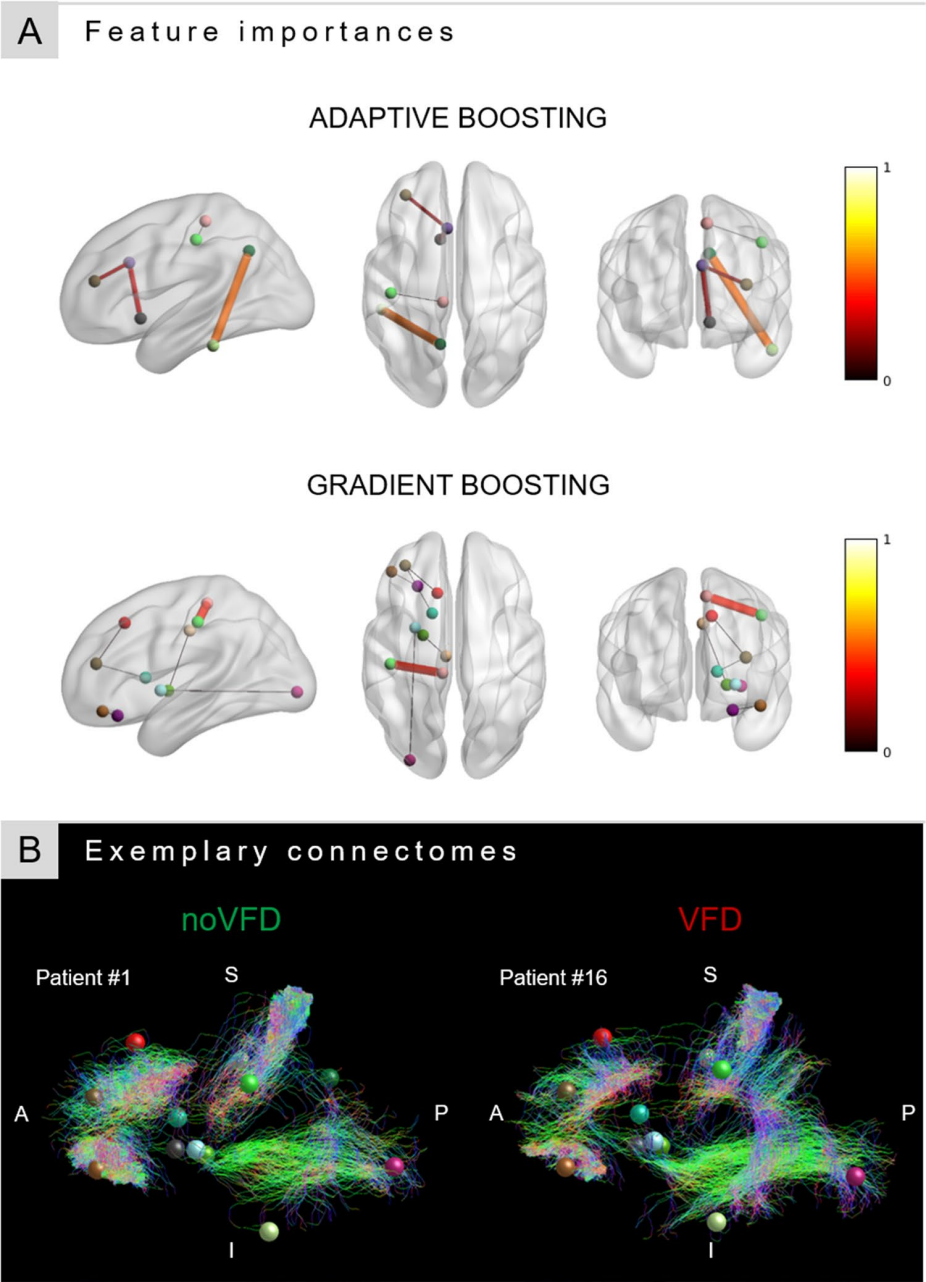
Features included in the ANN meta-classifier. (**A**) Specific post-hoc feature importances of the two boosting classifiers are visualized within the connectome. Both colour value and edge width indicate the respective Gini importance. (**B**) Exemplary streamline connectomes of the edges used by the ANN meta-classifier of a patient without (patient #1) and a patient with (patient #16) postsurgical VFD. Connectomes were downsampled to a total of 5000 streamlines for visualization purposes only.

In contrast to the individual classifiers, the ANN meta-classifier based on two boosting algorithms showed an increase in performance with a sensitivity of 95.24% and a specificity of 85.71%, yielding a total accuracy of 92.86%. Permutation testing confirmed a significant deviation from the performance on null distributions (*p* < 0.05). The post-hoc analysis of feature importances of the two boosting classifiers revealed, despite having the same individual classification accuracy, a different underlying weighting of connections. Specifically, the AdaBoost algorithm focused mainly on the connections inferior temporal gyrus to precuneus (Gini = 0.5), caudal anterior cingulate gyrus to nucleus accumbens (Gini = 0.28), and caudal anterior cingulate to rostral middle frontal gyrus (Gini = 0.2). In contrast to that, Gradient Tree Boosting showed a more widespread weighting of connections and a strong bias towards the connection between paracentral and postcentral gyrus (Gini = 0.41; see Fig. 5A). In exemplary connectomes, a lower streamline count in the connection from e.g. the inferior temporal gyrus to precuneus but a stronger overall connectivity in prefrontal areas can be observed in the patient without VFD compared to the exemplary patient with postsurgical VFD (see Fig. 5B).

In an additional explorative analysis, we added the information about the surgical procedure to the ANN meta-classifier. Using this additional information about the surgical planning further boosted the performance of the classifier to 96.43% accuracy. Other metrics can be found in the Supplementary Table S5.

## Discussion

In this study, we set out to find presurgical correlates of postsurgical VFD. While we here describe abundant postsurgical differences between patients with and without VFD in both gray and white matter structures, we could not find presurgical differences, neither on the level of voxels nor on the level of the respective structural networks. Despite the missing statistical significance, we could utilize supervised machine learning algorithms to extract patterns that seemingly distinguish these two patient groups purely based on presurgical structural connectomes with above-chance accuracies.

Imaging analyses of patients undergoing resective surgery in the temporal lobe have been repetitively performed^23–27^. This study is the first to globally relate gray and white matter sequelae of surgery to VFD. The structural changes observed are generally in line with results of these previous studies: both degeneration and neuroplastic reorganization following epilepsy surgery can occur and are mirrored by decrease or increase in gray matter volume or fractional anisotropy^28,29^. The voxel-based lesion-symptom mapping and correlation analyses allow to identify white matter changes associated with VFD. It should be added that these results may be correlates or causal links of the VFD. Most importantly the differentiation between causal links and correlates can only be undertaken on the basis of common knowledge about anatomy and physiology of the visual system. While the changes observed in the ML replicate the findings of previous studies and are somewhat expected, he bilateral nature of the VBM-cluster in the posterior division of both the ipsi- and the contralesional middle temporal gyri is somewhat surprising. This may be explained by *diaschisis*/secondary degeneration of so-called homotopic connectivity: *diaschisis* means the post-lesional change of brain structures remote from and connected with the anatomical site of damage^30^. *Homotopic connectivity* describes the special connectedness of mirror areas of the brain hemispheres^31^. This may also explain the white matter changes found in the external capsule where cortico-cortical association fibers are known to pass, which connect the two hemispheres. Accordingly, bihemispheric white matter changes have been identified previously as sequelae of temporal lobe surgery^24^. The possible reorganization, however, was not reflected in global network metrics based on the structural connectomes. The other surprising result pertains the location and form of the TBSS-cluster correlating with the degree of the VFD. The present study is the first to show changes in the course of optic radiation after sAH with an objective, i.e. ROI-independent, approach. However, it remains open to speculation why this cluster was found in the sagittal stratum and not more anterior in the temporal lobe. Possible reasons include the strong interindividual anatomical variability of the anterior part of the ML. The anatomical distance between the temporal pole and ML varies from 22 to 37 mm^32^. As the posterior part of the ML is not known to show the same degree of anatomical variability, only here diffusivity parameters could be related to the severity of VFD. Another possible explanation is the convergence of the anterior, central, and the posterior bundle of the optic radiation in the sagittal stratum leading to maximal fiber coherence, which makes the analysis of diffusivity parameters more sensitive. Interestingly, postoperative resection size and preoperative Euclidean distance between the temporal pole and the most anterior part of the ML did, in contrast to previous studies^17,22^, not serve as a predictor/correlate of VFD in the current study. Despite the relatively clear causal chain with the lesioning of ML, a clear consensus on which correlates or predictors are most valid has not been reached by previous studies, let alone has one marker been implemented in the clinical routine.

An important limitation of our study is the variability in the interval between timepoint of surgery and postoperative scan, ranging from 2 to 21 months. While we did not find any linear relationships between the surgery-scan-interval in neither VBM nor TBSS analysis and controlled for this variable in all pre- vs. postsurgical analyses, the possible non-linear effects of this variability are still unaccounted for. However, reducing this variability by excluding patients with a surgery-scan-interval greater than 12 months did not alter the overall results from our analyses (see Suppl. Fig. S1).

The discrepancy between the absence of presurgical structural correlates of VFDs and the seemingly successful prediction of VFDs using automated classifiers may seem contradictory, however, the difference between the outcome of statistical inference and statistical learning methods (the latter being classically evaluated using cross-validation) has been discussed multiple times^33,34^. Nevertheless, our explorative classification analysis, testing a multitude of *off-the-shelf* algorithms, should be treated with great caution. Specifically, the use of a *leave-one-out* cross-validation scheme in small datasets is thoroughly discussed in the current neuroimaging literature^35^. It was shown that already minimal changes in the design of a supervised classifier can lead to so-called *vibration effects* which manifest in large differences in the standard performance evaluation metrics. Therefore, the real generalization performance of a classifier cannot be reliably approximated in small datasets as the one reported in this study. To give some information about the reliability of our classifiers, we utilized a permutation test randomly swapping the group labels and evaluating their performance on each permutation. The outcomes of this test already show that especially the simpler classifiers fail to extract real group differences for classification indicating instable generalization performances. On the same note, another big limitation of our classification approach is the strong class imbalance of our dataset. Only seven of the 28 patients in our study showed no postsurgical VFD. A trivial classifier, always predicting postsurgical VFD irrespective of the input, would thereby already result in an accuracy of 75%. Several of the individual classifiers, such as k-nearest neighbours, Naïve Bayes or Random Forest, show exactly this kind of vulnerability for the dominance of the VFD class. However, especially the adaptive and gradient boosting algorithms, developed to overcome this kind of dataset bias, seem to find a more balanced decision boundary between the two classes and reach a higher discriminatory power, also reflected in the different distribution of feature importances over individual connections. The stacking of these by a simple ANN as meta-classifier seemingly combines the best of both worlds. Lastly, feeding this classifier with information about the surgical procedure further boosts its performance. This near-perfect performance, however, can be deceptive and should be, additionally to the already mentioned limitations, seen in context with the unbalanced number of patients undergoing transsylvian and subtemporal amygdalohippocampectomy. While this is unfortunate in the context of our analyses, it results from the challenge of prospectively acquiring data in a clinical setting. Therefore, taking together both the size and the class-imbalance of the dataset, the generalizability of even the best performing classifier in this study cannot be guaranteed and should therefore be understood as mere explorative analysis that may a hint towards a possible predictive approach in the future, that needs to be developed in a larger, balanced sample and validated on yet another, external dataset.

## Methods

### Study design and patient cohort

Twenty-eight patients with pharmacoresistant mesial temporal lobe epilepsy were prospectively included in our study (mean age ± SD: 38.82 ± 12.80; 15 females). All patients underwent presurgical assessment at the Department of Epileptology followed by a sAH at the Department of Neurosurgery of the University Hospital Bonn between 2009 and 2012. Ten patients were operated using a subtemporal access and 18 patients using a transsylvian access for clinical reasons. T1 and DTI scans were acquired one day before and several months after (mean interval ± SD: 6.6 ± 5.3 months) the surgical procedure. At the same days of the pre- and postoperative scans, the presence and severity of VFD was assessed using a Twinfield perimeter (Oculus Inc., Wetzlar, Germany), which is able to perform automated static and kinetic perimetry according to the Goldmann standard at the Department of Ophthalmology of the University Hospital Bonn. See Table 2 for details. Additional to the patient cohort, T1 and DTI scans of 32 healthy controls (mean age ± SD: 32.52 ± 6.70; 16 females) were acquired. This study and all its experimental protocols were approved by the Institutional Review Board of the medical faculty of the University of Bonn. All methods were performed in accordance with the guidelines and regulations of this ethics board and in accordance with the Declaration of Helsinki. Informed consent was obtained from all participants and/or their legal guardians. The data that support the findings of this study are available on request from the corresponding author. Datasets are not publicly available as they contain information that could comprise the privacy of research participants.

**Table 2:** Demographical and clinical characteristics of the patient group.

ID	Age at onset of seizures (years)	Age at surgery (years)	Surgery–post-scan–interval (months)	Neurosurgical approach	Side	Preoperative VFD	Postoperative VFD	Postoperative driving ability	ILAE outcome (last follow-up)
1	1–5	16–20	21	Transsylv	R	0	1	No	5
2	11–15	16–20	4	Transsylv	R	0	1	Yes	1
3	16–20	41–45	19	Subtemp	L	0	0	Yes	4
4	21–25	46–50	12	Transsylv	L	1	1	No	1
5	1–5	31–35	11	Subtemp	R	0	0	Yes	1
6	11–15	51–55	10	Subtemp	L	0	2	No	3
7	1–5	46–50	6	Subtemp	L	0	0	Yes	1
8	11–15	31–35	16	Transsylv	L	0	2	No	1
9	1–5	41–45	5	Transsylv	L	0	2	No	1
10	1–5	46–50	5	Transsylv	R	0	1	Yes	1
11	26–30	46–50	11	Transsylv	L	0	2	No	1
12	26–30	31–35	3	Transsylv	L	0	2	No	5
13	1–5	21–25	4	Transsylv	L	0	1	Yes	3
14	1–5	41–45	3	Transsylv	R	0	1	Yes	1
14	16–20	46–50	3	Subtemp	L	2	0	Yes	4
16	31–35	71–75	3	Transsylv	L	0	2	No	1
17	31–35	36–40	3	Transsylv	R	1	3	No	4
18	11–15	51–55	3	Subtemp	L	0	1	Yes	1
19	16–20	26–30	3	Transsylv	L	0	1	No	1
20	21–25	26–30	6	Transsylv	R	0	3	No	3
21	36–40	36–40	3	Transsylv	L	0	1	No	1
22	26–30	31–35	2	Transsylv	L	0	1	Yes	1
23	11–15	21–25	5	Transsylv	R	1	1	No	1
24	36–40	36–40	11	Subtemp	L	0	0	Yes	1
25	6–10	36–40	3	Transsylv	R	0	3	No	1
26	21–25	61–65	4	Subtemp	L	2	3	No	3
27	11–15	26–30	3	Subtemp	L	0	0	Yes	3
28	16–20	26–30	2	Subtemp	R	0	0	Yes	1

Classification of visual field deficit: 0 = no anopia, 1 = incomplete homonymous quadrantopia, 2 = complete homonymous quadrantopia, 3 = incomplete homonymous hemianopia. (L = left, R = Right, subtemp = subtemporal access, transsylv = transsylvian access).

### Image acquisition and preprocessing

For all patients pre- and postsurgical T1 and DTI scans were acquired using a Siemens Magnetom Trio (3 T) MRI-scanner. Scans were acquired with an 8-channel head receive coil. We ran a 3D T1-weighted anatomical sequence (resolution = 1.0 × 1.0 × 1.0 mm, TR = 1570 ms, TE = 3.42 ms, flip angle = 15°) and a diffusion-weighted single shot dual echo spin-echo echo planar imaging sequence (resolution = 1.72 × 1.72 × 1.7 mm^3^, TR = 12,000 ms, TE = 100 ms, flip angle = 90°) with 60 directions and a b-value of 1000 s/mm^2^ as well as six baseline scans with a b-value of 0 s/mm^2^. Preprocessing of T1 and DTI datasets was realized using FMRIB’s Software Library 5.0 (FSL)^36^ and the Tolerably Obsessive Registration and Tensor Optimization Indolent Software Ensemble (TORTOISE)^37^. T1 scans were skull-stripped and corrected for b0 field inhomogeneities. DTI scans were corrected for susceptibility-induced geometric distortions using a constraint registration approach^38^. In the same registration step, DTI scans were corrected for within-subject motion and eddy-currents, keeping interpolation effects minimal. Finally, a diffusion tensor model was fitted voxel-wise and fractional anisotropy (FA) values were calculated. For all following analyses, ipsilesional hemispheres were flipped to the same side.

### Voxel-based lesion-symptom mapping

To analyze the tissue-independent influence of the specific lesion extent and location on the degree of VFD, we conducted a voxel-based lesion-symptom mapping (VLSM) analysis using the nonparametric mapping toolbox (NPM) implemented in MRIcron version 8^39^. For all patients, lesion masks of the resection cavity were manually demarcated on the T1. Based on the individual lesion masks a colour-coded overlay map of all affected voxels across patients was calculated to provide an overview of the lesion extents (Fig. 1A). This was achieved by normalizing each patient’s presurgical T1 scan to the MNI152 template using non-linear registrations^36^. These non-linear warp fields were in turn combined with matrices resulting of the linear registration of the postsurgical to the presurgical T1 scan and applied to the individual lesion masks. The normalized lesion masks were averaged and thresholded at 20%. Based on this normalization, the lesion masks were included in the voxel-based lesion-symptom mapping analysis. Here, we used a permutation-based Brunner–Munzel rank test to analyze the statistical contribution of each lesion voxel on the postsurgical binocular results of the automated perimetry. For valid statistical inference, only voxels affected in at least 10 patients were considered^40^. Results were corrected for multiple comparisons using a permutation-based family-wise error (FWE) correction. Clusters were considered significant at FWE-corrected *p* < 0.05.

### Voxel-based morphometry

To analyze possible GM correlates of VFD the pre- and postsurgical T1 scans were entered into a voxel-based morphometry (VBM) analysis using the optimized FSL-VBM protocol^41^. First, the skull-stripped structural scans were segmented into GM partial volume estimates which were in turn non-linearly registered to the MNI152 standard space. For the computation of the postsurgical normalization warp fields the individual lesions were masked out, leading to a registration solely based on the intact brain tissue. From the resulting pre- and postsurgical normalized images of all patients, a study-specific and left–right symmetric brain template was calculated. All scans in native space were then registered to this template using again non-linear transformations and lesion masking. This step included the multiplication of the normalized partial volume estimates by the Jacobian of their respective warp field, to correct for local expansions and contractions due to the non-linear component of the registration. These modulated GM partial volume estimates were then smoothed using an isotropic three-dimensional Gaussian kernel with a sigma of 3 mm. Permutation-based threshold-free cluster enhancement was applied to statistically evaluate GM differences between pre- and postsurgical scans (paired *t*-test) and correct for multiple comparisons^42^. All statistical models were adjusted for *between-scan interval*, *age at scan, surgery-scan interval,* and *TLE-laterality*.

### Tract-based spatial statistics

We conducted a tract-based spatial statistics analysis (TBSS) of patients’ pre- and postsurgical FA maps to identify possible WM correlates of VFDs^43^. All FA maps were registered to the MNI152 standard space using non-linear transformations. Precise normalization of postsurgical maps was achieved by masking lesional voxels in the computation of the warp fields. A mean FA image was created, masked with the canonical lesion mask (see Fig. 1A) and thinned, resulting in a mean FA skeleton representing the centers of all WM pathways common to the patient group. Each patient’s aligned FA maps were then projected on to the mean skeleton mask and in turn used in a permutation-based statistical analysis. Parallel to the above described VBM analyses, statistical models comparing pre- and postsurgical scans as well as linear relationships between WM differences and VFD degree were implemented using two-dimensional threshold-free cluster enhancement and adjusted for *between-scan interval*, *age at scan*, *surgery-scan interval*, and *TLE-laterality*. To inform anatomical localization of the results, the optic radiation was reconstructed by means of probabilistic fiber tractography in every patient and a canonical optic radiation was generated (see Supplementary Material for a detailed description).

### Node delineation

To estimate a structural connectome, we needed to delineate respective network nodes. For this purpose, we parcellated the presurgical T1-scans of all patients into 84 regions according to the Desikan-Killiany atlas using the standard FreeSurfer (version 5.0) processing stream^44^. Individual parcellations were visually controlled and, if needed, manually corrected. To delineate the same nodes in the postsurgical scans, manual lesion masks were constructed and presurgical brain parcellations were linearly registered to the respective postsurgical T1 volume. To account for possible registration biases due to postsurgical brain distortions, we utilized a robust registration method for longitudinal datasets to transform both pre- and postsurgical images to their midspace^45^. Thereby, the registration is not biased towards either the pre- or postsurgical scan. Registered parcellations were in turn multiplied by the inverted lesion mask, leaving us with postsurgical network nodes excluding the individual lesion. In a second step, both pre- and postsurgical nodes had to be linearly registered to the diffusion space. To ensure an accurate anatomical correspondence between T1 and DTI volumes, we calculated a mean b0 image, inverted its contrast and, finally, used boundary-based linear registration to translate our anatomical nodes to diffusion space^46,47^. All registration steps were controlled visually.

### Structural connectome processing

It is known that the classical diffusion tensor model fails to adequately model the abundant regions of multiple fiber orientations in the brain, which in turn negatively affects the structural connectome construction^48–50^. For this reason, we constructed the connectivity-based structural connectome using a higher order diffusion model as implemented by MRtrix3^51^. First, a regular diffusion tensor model was fitted voxelwise for the whole DTI volume and fractional anisotropy (FA) values were calculated. Voxels showing the highest FA (> 0.7, i.e. being the closest to a ‘single fiber’ voxel) determined the response function which in turn was used for the constrained spherical deconvolution of the diffusion-weighted signal estimating the specific fiber-orientation distribution (FOD) for every voxel^52,53^. Based on the voxelwise FODs a whole-brain probabilistic streamline tractography could be conducted. To mitigate the effect of spurious fiber trajectories in GM regions, we used “anatomically-constrained tractography” by segmenting the GM-WM interface as seed- and termination mask in diffusion space from the previously described parcellations of the T1 scans^54^. From this mask, 10,000,000 streamlines were dynamically seeded and progressed using a second-order integration over the FODs^55^. Further tracking parameters included a step size of 0.85 mm, minimum streamline length of 4 mm, maximum length of 250 mm and a FOD amplitude cut-off value of 0.05. Ensuring the robustness of streamline estimates, whole-brain tractograms were selectively filtered by a factor of 0.5, concluding tractograms of 5,000,000 streamlines each^56^. Finally, seed and termination points were mapped onto the parcellated network nodes and streamline counts between nodes were taken as edge weights of the connectivity graphs.

### Connectivity-based statistics

Global differences in connectivity-graphs were assessed using the Network-based statistics toolbox^57^. Longitudinal comparisons of pre- and postsurgical networks were tested using paired *t*-tests. To avoid the report of obvious differences between pre- and postsurgical connections to and from amygdala and hippocampus, all connections of these regions were zeroed in presurgical connectomes as well. Significance was evaluated using network-based statistics including non-parametric permutation testing with 10,000 permutations to correct for the family-wise error rate. Results were considered significant at a FWE-corrected *p*-value below 0.05.

### Graph-theoretic measures

To quantify and compare global network characteristics between patients with and without postsurgical VFD, we calculated graph-theoretic network measures using the Brain Connectivity Toolbox. The following global network-metrics were considered: (1) Average degree (describing the average number of links connected to a node), (2) average clustering coefficient (average number of triangles around nodes), (3) transitivity (normalized clustering coefficient for the individual degree of nodes), (4) density (fraction of present connections to possible connections), (5) routing efficiency (average inverse shortest path length), (6) assortativity (tendency of nodes to link to nodes with a similar degree), (7) coreness (amount of subgraphs comprising of nodes with the same degree), (8) average node betweennes centrality (average fraction of all shortest paths containing a node), and (9) small-worldness (transitivity over the average shortest path length). Network measures were chosen to describe global features between the networks under consideration, namely the functional integration and segregation of the networks as well as the network resilience to lesions. For mathematical formulations and interpretations the interested reader is referred to the review by Rubinov and Sporns^58^. Group comparisons of network measures were conducted using two-tailed unpaired *t*-tests. Statistical inference was confirmed using bootstrap analyses with 10.000 times resampling.

### Connectome-based classification

As an additional explorative analysis, we trained five of the most common supervised classification algorithms on the connectomes of the lesioned hemisphere using their default implementation in scikit-learn^59^: (1) k-nearest neighbors, (2) Gaussian Naïve Bayes, (3) logistic regression, (4) Decision Tree, and (5) Support Vector Machine. As our sample is unbalanced and small compared to typical statistical learning problems, we furthermore included four tree-based ensemble and boosting classifiers: (6) Random Forests, (7) Extremely Randomized Trees, (8) Gradient Tree Boosting, and (9) AdaBoost. Ensemble methods and boosting can be favorable for this type of datasets, as here weak (Decision Tree) learners are iteratively trained on the dataset and finally combined to a strong learner. After each added weak learner, the weight of misclassified samples gets increased and forcing the next learner to focus more on those samples, thereby counteracting the dominance of one class in the dataset^60,61^. The generalizability of all classifiers was evaluated using a *Leave-One-Out *cross-validation scheme. We statistically compared the individual classification performances to the classifiers’ performance on 10,000 null distributions using permutation testing. Furthermore, to ensure that classifiers do not use spurious structural connections, all classifiers were trained on a sparse connectome including only connections with a minimum streamline count of 10 in all patients.

### Meta classifier construction

To increase overall capacity of our presurgical classification procedure, we combined the two boosting classifiers in an additional ensemble learner by means of stacking. Here, the two base-classifiers were trained on our original presurgical sparse lesioned connectomes. A supervised meta-classifier in turn was added to integrate the outputs of the two classifiers as meta-features during training. Our meta-classifier in this case was a backpropagating artificial neural network (ANN) comprising of two hidden layers with 100 and 50 neurons using a rectified linear unit activation function and two sigmoid output neurons. Like the other supervised classifiers, our experimental stacking classifier was trained on the data using *Leave-One-Out* cross-validation and performance scores were statistically compared to 10,000 null distributions constructed using permutation testing. Furthermore, a post-hoc analysis of Gini importance according to the two boosting classifiers was conducted to visualize the specific connections utilized in the classification.

## Supplementary Information

Supplementary Information.

## References

[1] Semah F (1998). Is the underlying cause of epilepsy a major prognostic factor for recurrence?. Neurology.

[2] Thijs RD, Surges R, O’Brien TJ, Sander JW (2019). Epilepsy in adults. Lancet.

[3] Semah F, Ryvlin P (2005). Can we predict refractory epilepsy at the time of diagnosis?. Epileptic Disord..

[4] Engel J (2012). Early surgical therapy for drug-resistant temporal lobe epilepsy: A randomized trial. JAMA.

[5] Wiebe S, Blume WT, Girvin JP, Eliasziw M, Effectiveness and Efficiency of Surgery for Temporal Lobe Epilepsy Study Group (2001). A randomized, controlled trial of surgery for temporal-lobe epilepsy. N. Engl. J. Med..

[6] Jain P, Tomlinson G, Snead C, Sander B, Widjaja E (2018). Systematic review and network meta-analysis of resective surgery for mesial temporal lobe epilepsy. J. Neurol. Neurosurg. Psychiatry.

[7] Yaşargil MG, Teddy PJ, Roth P (1985). Selective amygdalo-hippocampectomy. Operative anatomy and surgical technique. Adv. Tech. Stand. Neurosurg..

[8] Hori T (1993). Subtemporal amygdalohippocampectomy for treating medically intractable temporal lobe epilepsy. Neurosurgery.

[9] Schmeiser B (2017). Transsylvian selective amygdalohippocampectomy for mesiotemporal epilepsy: Experience with 162 procedures. Neurosurgery.

[10] Lutz MT, Clusmann H, Elger CE, Schramm J, Helmstaedter C (2004). Neuropsychological outcome after selective amygdalohippocampectomy with transsylvian versus transcortical approach: A randomized prospective clinical trial of surgery for temporal lobe epilepsy. Epilepsia.

[11] Winston GP (2013). Epilepsy surgery, vision, and driving: what has surgery taught us and could modern imaging reduce the risk of visual deficits?. Epilepsia.

[12] Jeelani NUO (2010). ‘Hemispherical asymmetry in the Meyer’s Loop’: A prospective study of visual-field deficits in 105 cases undergoing anterior temporal lobe resection for epilepsy. J. Neurol. Neurosurg. Psychiatry.

[13] Yeni SN (2008). Visual field defects in selective amygdalohippocampectomy for hippocampal sclerosis: the fate of Meyer’s loop during the transsylvian approach to the temporal horn. Neurosurgery.

[14] van Lanen RHGJ (2018). Visual field deficits after epilepsy surgery: A new quantitative scoring method. Acta Neurochir..

[15] Delev D (2016). Vision after trans-sylvian or temporobasal selective amygdalohippocampectomy: A prospective randomised trial. Acta Neurochir..

[16] Jäncke L (2009). The plastic human brain. Restor. Neurol. Neurosci..

[17] Yogarajah M (2009). Defining Meyer’s loop–temporal lobe resections, visual field deficits and diffusion tensor tractography. Brain.

[18] Winston GP (2012). Optic radiation tractography and vision in anterior temporal lobe resection. Ann. Neurol..

[19] Winston GP (2011). Diffusion tensor imaging tractography to visualize the relationship of the optic radiation to epileptogenic lesions prior to neurosurgery. Epilepsia.

[20] de Souza JPSAS (2019). Fractional anisotropy of the optic radiations correlates with the visual field after epilepsy surgery. Neuroradiology.

[21] Chen X, Weigel D, Ganslandt O, Buchfelder M, Nimsky C (2009). Prediction of visual field deficits by diffusion tensor imaging in temporal lobe epilepsy surgery. Neuroimage.

[22] Powell HWR (2005). MR tractography predicts visual field defects following temporal lobe resection. Neurology.

[23] Yogarajah M (2010). The structural plasticity of white matter networks following anterior temporal lobe resection. Brain.

[24] Schoene-Bake J-C (2009). Widespread affections of large fiber tracts in postoperative temporal lobe epilepsy. NeuroImage.

[25] Winston GP, Stretton J, Sidhu MK, Symms MR, Duncan JS (2014). Progressive white matter changes following anterior temporal lobe resection for epilepsy. NeuroImage.

[26] Yasuda CL (2010). Dynamic changes in white and gray matter volume are associated with outcome of surgical treatment in temporal lobe epilepsy. NeuroImage.

[27] McDonald CR (2010). Changes in fiber tract integrity and visual fields after anterior temporal lobectomy. Neurology.

[28] Buetefisch CM (2015). Role of the contralesional hemisphere in post-stroke recovery of upper extremity motor function. Front. Neurol..

[29] Dodd KC, Nair VA, Prabhakaran V (2017). Role of the contralesional vs ipsilesional hemisphere in stroke recovery. Front. Hum. Neurosci..

[30] Seitz RJ (1999). The role of diaschisis in stroke recovery. Stroke.

[31] Mancuso L (2019). The homotopic connectivity of the functional brain: A meta-analytic approach. Sci. Rep..

[32] Ebeling U, Reulen H-J (1988). Neurosurgical topography of the optic radiation in the temporal lobe. Acta Neurochir..

[33] Bzdok D (2017). Classical Statistics and Statistical Learning in Imaging Neuroscience. Front. Neurosci..

[34] Bzdok D, Altman N, Krzywinski M (2018). Statistics versus machine learning. Nat. Methods.

[35] Varoquaux G (2018). Cross-validation failure: Small sample sizes lead to large error bars. NeuroImage.

[36] Jenkinson M, Beckmann CF, Behrens TEJ, Woolrich MW, Smith SM (2012). FSL. Neuroimage.

[37] Pierpaoli, C. *et al.* TORTOISE: An integrated software package for processing of diffusion MRI data. *Conference proceedings* (2010).

[38] Yao, X.-F. & Song, Z.-J. Deformable Registration for Geometric Distortion Correction of Diffusion Tensor Imaging. in *Computer Analysis of Images and Patterns* (eds. Real, P., Diaz-Pernil, D., Molina-Abril, H., Berciano, A. & Kropatsch, W.) 545–553 (Springer, New York, 2011). 10.1007/978-3-642-23672-3_66.

[39] Rorden C, Karnath H-O, Bonilha L (2007). Improving lesion-symptom mapping. J. Cogn. Neurosci..

[40] Medina J, Kimberg DY, Chatterjee A, Coslett HB (2010). Inappropriate usage of the Brunner–Munzel test in recent voxel-based lesion-symptom mapping studies. Neuropsychologia.

[41] Douaud G (2009). Schizophrenia delays and alters maturation of the brain in adolescence. Brain.

[42] Winkler AM, Ridgway GR, Webster MA, Smith SM, Nichols TE (2014). Permutation inference for the general linear model. Neuroimage.

[43] Smith SM (2006). Tract-based spatial statistics: Voxelwise analysis of multi-subject diffusion data. Neuroimage.

[44] Fischl B (2002). Whole brain segmentation: Automated labeling of neuroanatomical structures in the human brain. Neuron.

[45] Reuter M, Rosas HD, Fischl B (2010). Highly accurate inverse consistent registration: A robust approach. NeuroImage.

[46] Bhushan C (2015). Co-registration and distortion correction of diffusion and anatomical images based on inverse contrast normalization. Neuroimage.

[47] Greve DN, Fischl B (2009). Accurate and robust brain image alignment using boundary-based registration. Neuroimage.

[48] Behrens TEJ, Berg HJ, Jbabdi S, Rushworth MFS, Woolrich MW (2007). Probabilistic diffusion tractography with multiple fibre orientations: What can we gain?. Neuroimage.

[49] Sotiropoulos, S. N. & Zalesky, A. Building connectomes using diffusion MRI: Why, how and but. *NMR Biomed* **32** (2019). 10.1002/nbm.3752 PMC6491971 28654718

[50] Tournier J-D, Mori S, Leemans A (2011). Diffusion tensor imaging and beyond. Magn. Reson. Med..

[51] Tournier J-D (2019). MRtrix3: A fast, flexible and open software framework for medical image processing and visualisation. NeuroImage.

[52] Tournier J-D, Calamante F, Connelly A (2007). Robust determination of the fibre orientation distribution in diffusion MRI: Non-negativity constrained super-resolved spherical deconvolution. NeuroImage.

[53] Tournier J-D, Calamante F, Connelly A (2013). Determination of the appropriate b value and number of gradient directions for high-angular-resolution diffusion-weighted imaging. NMR Biomed..

[54] Smith RE, Tournier J-D, Calamante F, Connelly A (2012). Anatomically-constrained tractography: Improved diffusion MRI streamlines tractography through effective use of anatomical information. NeuroImage.

[55] Smith RE, Tournier J-D, Calamante F, Connelly A (2015). SIFT2: Enabling dense quantitative assessment of brain white matter connectivity using streamlines tractography. NeuroImage.

[56] Smith RE, Tournier J-D, Calamante F, Connelly ASIFT (2013). Spherical-deconvolution informed filtering of tractograms. NeuroImage.

[57] Zalesky A, Fornito A, Bullmore ET (2010). Network-based statistic: Identifying differences in brain networks. NeuroImage.

[58] Rubinov M, Sporns O (2010). Complex network measures of brain connectivity: Uses and interpretations. NeuroImage.

[59] Pedregosa F (2011). Scikit-learn: Machine learning in Python. J. Mach. Learn. Res..

[60] Schapire RE (1990). The strength of weak learnability. Mach. Learn..

[61] Freund, Y. & Schapire, R. E. A desicion-theoretic generalization of on-line learning and an application to boosting. in *Computational Learning Theory* (ed. Vitányi, P.) 23–37 (Springer, New York, 1995). 10.1007/3-540-59119-2_166.

