# Supplementary material for: Multi-scale image analysis and prediction of visual field defects after selective amygdalohippocampectomy
Source: Sci Rep. 2021 Jan 14;11:1444. doi: 10.1038/s41598-020-80751-x (PMC7809286; doi:10.1038/s41598-020-80751-x)
Supplement: Supplementary file 1 — Supplementary Information. [file 41598_2020_80751_MOESM1_ESM.docx]

**Title:** Multi-scale image analysis and prediction of visual field defects after selective amygdalohippocampectomy

**Authors:** Bastian David^1^, Jasmine Eberle^1,2^, Daniel Delev^3^, Jennifer Gaubatz^1^, Conrad Prillwitz^1^, Jan Wagner^4^, Jan-Christoph Schoene-Bake^5^, Guido Luechters^6^, Alexander Radbruch^7^, Bettina Wabbels^8^, Johannes Schramm^9^, Bernd Weber^10^, Rainer Surges^1^, Christian Elger^1^, Theodor Rüber* ^1,11,12^

**Affiliations:** ^1^Department of Epileptology, University Hospital Bonn, Bonn, Germany; ^2^Clinic for Neurology and Palliative Medicine, Municipal Hospital Köln-Merheim, Cologne, Germany; ^3^Department of Neurosurgery, RWTH University Aachen, Aachen, Germany; ^4^Department of Neurology, University of Ulm and Universitäts- and Rehabilitationskliniken Ulm, Ulm, Germany; ^5^Department of Pediatric Kidney, Liver and Metabolic Diseases, Hannover Medical School, Hannover, Germany; ^6^Center for Development Research, University of Bonn; ^7^Department of Neuroradiology, University Hospital Bonn, Bonn, Germany; ^8^Department of Ophthalmology, University Hospital Bonn, Bonn, Germany; ^9^Medical Faculty, University Hospital Bonn, Bonn, Germany; ^10^Institute of Experimental Epileptology and Cognition Research, University Hospital Bonn, Bonn, Germany; ^11^Epilepsy Center Frankfurt Rhine-Main, Department of Neurology, Goethe University Frankfurt, Frankfurt am Main, Germany; ^12^Center for Personalized Translational Epilepsy Research (CePTER), Goethe-University Frankfurt, Frankfurt am Main, Germany

**Corresponding author:**

Theodor Rüber, MD

Department of Epileptology, University of Bonn Medical Center

Sigmund-Freud-Str. 25

53127 Bonn

Germany

Email: theodor.rueber@ukbonn.de

Phone: +49228-6885264

**Supplementary Material**

*Tractography of the Meyer’s loop and canonical tracts*

Probabilistic tractography of the optic radiation including the Meyer`s Loop was carried out using probtrackx2 from FSL (version 5.0). Regions of interest (ROIs) were drawn manually on subject-specific FA maps in native space by one rater (J.E.). Additionally, an overlaid principal diffusion direction map was used to ameliorate inter-subject consistency. As a seed region, an 18-voxel ROI was defined at the lateral geniculate nucleus (LGN) on two axial slices. Two waypoint masks were created at the lateral wall of the occipital horn of the lateral ventricle on one coronal slice and in the primary visual cortex in a sagittal plane eight voxels lateral to the midline.^1^ In order to remove artefactual connections, a midline exclusion mask was defined. In addition, a second coronal exclusion mask was generated by a previously described iterative process: fiber tracts were generated from the seed region and an exclusion mask, which – at first located twenty voxels more anterior than the temporal horn – was moved posteriorly by one voxel per iteration until a reduction in tract volume of ≥10% was reached. At this point, a visible thinning of the estimated trajectory of the optic radiation was observable. The generated tract was thresholded to include only those voxels with a tract intensity of more than 5%. As a result, tracts corrected for artefactual connections could be created in all subjects both ipsi- and contralesional in pre- and postoperative scans.^2^ All individual ipsilesional tracts in FA space were aligned and normalized to the MNI T1 1mm template using the non-lineal transformation matrices of the FA maps (see *Tract-based spatial statistics* in Methods in the body of the manuscript). Canonical tracts were obtained by summing up the normalized tracts to one main tract. This tract was then thresholded by 0.5.

*References*

1. Winston, G. P. *et al.* Diffusion tensor imaging tractography to visualize the relationship of the optic radiation to epileptogenic lesions prior to neurosurgery. *Epilepsia* **52**, 1430–1438 (2011).

2. Yogarajah, M. *et al.* Defining Meyer’s loop-temporal lobe resections, visual field deficits and diffusion tensor tractography. *Brain* **132**, 1656–1668 (2009).

**Table S 1: No VFD pre- vs postsurgical connectivity strength differences.** Edges showing a significant postsurgical (vs. presurgical) decrease in connectivity strength in patients without VFD (FWE-corr. p < 0.05). Abbreviations follow the standard FreeSurfer lookup table.

| Edges (node to node) |
| --- |
| ctx-lh-entorhinal - ctx-lh-parahippocampal |
| ctx-lh-inferiortemporal - ctx-lh-parahippocampal |
| ctx-lh-parahippocampal - ctx-lh-insula |
| ctx-lh-temporalpole - Left-Pallidum |

**Table S 2:** **VFD pre- vs postsurgical connectivity strength differences.** Edges showing a significant postsurgical (vs. presurgical) decrease in connectivity strength in patients with VFD (FWE-corr. p < 0.05). Abbreviations follow the standard FreeSurfer lookup table.

| Edges (node to node) | Edges (node to node) |
| --- | --- |
| ctx-lh-entorhinal - ctx-lh-isthmuscingulate | ctx-lh-lateralorbitofrontal - ctx-lh-insula |
| ctx-lh-fusiform - ctx-lh-isthmuscingulate | ctx-lh-lingual - ctx-lh-insula |
| ctx-lh-fusiform - ctx-lh-lateralorbitofrontal | ctx-lh-middletemporal - ctx-lh-insula |
| ctx-lh-inferiortemporal - ctx-lh-lateralorbitofrontal | ctx-lh-parahippocampal - ctx-lh-insula |
| ctx-lh-entorhinal - ctx-lh-lingual | ctx-lh-parsorbitalis - ctx-lh-insula |
| ctx-lh-fusiform - ctx-lh-medialorbitofrontal | ctx-lh-superiortemporal - ctx-lh-insula |
| ctx-lh-inferiortemporal - ctx-lh-medialorbitofrontal | ctx-lh-temporalpole - ctx-lh-insula |
| ctx-lh-lateralorbitofrontal - ctx-lh-middletemporal | ctx-lh-fusiform - Left-Thalamus-Proper |
| ctx-lh-entorhinal - ctx-lh-parahippocampal | ctx-lh-inferiortemporal - Left-Thalamus-Proper |
| ctx-lh-fusiform - ctx-lh-parsopercularis | ctx-lh-superiortemporal - Left-Thalamus-Proper |
| ctx-lh-fusiform - ctx-lh-parsorbitalis | ctx-lh-temporalpole - Left-Thalamus-Proper |
| ctx-lh-inferiortemporal - ctx-lh-parsorbitalis | ctx-lh-transversetemporal - Left-Thalamus-Proper |
| ctx-lh-entorhinal - ctx-lh-parstriangularis | ctx-lh-fusiform - Left-Caudate |
| ctx-lh-inferiortemporal - ctx-lh-parstriangularis | ctx-lh-parsorbitalis - Left-Caudate |
| ctx-lh-fusiform - ctx-lh-rostralanteriorcingulate | ctx-lh-superiortemporal - Left-Caudate |
| ctx-lh-inferiortemporal - ctx-lh-rostralanteriorcingulate | ctx-lh-entorhinal - Left-Putamen |
| ctx-lh-entorhinal - ctx-lh-rostralmiddlefrontal | ctx-lh-fusiform - Left-Putamen |
| ctx-lh-fusiform - ctx-lh-rostralmiddlefrontal | ctx-lh-inferiortemporal - Left-Putamen |
| ctx-lh-entorhinal - ctx-lh-superiorfrontal | ctx-lh-lingual - Left-Putamen |
| ctx-lh-fusiform - ctx-lh-superiorfrontal | ctx-lh-middletemporal - Left-Putamen |
| ctx-lh-entorhinal - ctx-lh-superiortemporal | ctx-lh-parahippocampal - Left-Putamen |
| ctx-lh-fusiform - ctx-lh-superiortemporal | ctx-lh-parsorbitalis - Left-Putamen |
| ctx-lh-inferiortemporal - ctx-lh-superiortemporal | ctx-lh-superiortemporal - Left-Putamen |
| ctx-lh-lateralorbitofrontal - ctx-lh-superiortemporal | ctx-lh-temporalpole - Left-Putamen |
| ctx-lh-lingual - ctx-lh-superiortemporal | ctx-lh-entorhinal - Left-Pallidum |
| ctx-lh-medialorbitofrontal - ctx-lh-superiortemporal | ctx-lh-fusiform - Left-Pallidum |
| ctx-lh-parahippocampal - ctx-lh-superiortemporal | ctx-lh-inferiortemporal - Left-Pallidum |
| ctx-lh-parsorbitalis - ctx-lh-superiortemporal | ctx-lh-lateraloccipital - Left-Pallidum |
| ctx-lh-rostralanteriorcingulate - ctx-lh-superiortemporal | ctx-lh-middletemporal - Left-Pallidum |
| ctx-lh-entorhinal - ctx-lh-temporalpole | ctx-lh-superiortemporal - Left-Pallidum |
| ctx-lh-lingual - ctx-lh-temporalpole | ctx-lh-temporalpole - Left-Pallidum |
| ctx-lh-parahippocampal - ctx-lh-temporalpole | ctx-lh-transversetemporal - Left-Pallidum |
| ctx-lh-parsorbitalis - ctx-lh-temporalpole | ctx-lh-inferiortemporal - Left-Accumbens-area |
| ctx-lh-parahippocampal - ctx-lh-transversetemporal | ctx-lh-superiortemporal - ctx-rh-pericalcarine |
| ctx-lh-entorhinal - ctx-lh-insula | ctx-lh-inferiortemporal - ctx-rh-superiorfrontal |
| ctx-lh-fusiform - ctx-lh-insula | ctx-lh-fusiform - ctx-rh-superiortemporal |
| ctx-lh-inferiortemporal - ctx-lh-insula |  |

**Table S 3: Subtemporal sAH pre- vs postsurgical connectivity strength differences.** Edges showing a significant postsurgical (vs. presurgical) decrease in connectivity strength in patients who underwent subtemporal sAH (FWE-corr. p < 0.05). Abbreviations follow the standard FreeSurfer lookup table.

| Edges (node to node) |
| --- |
| ctx-lh-entorhinal - ctx-lh-fusiform |
| ctx-lh-entorhinal - ctx-lh-lingual |
| ctx-lh-entorhinal - ctx-lh-parahippocampal |
| ctx-lh-fusiform - ctx-lh-parahippocampal |
| ctx-lh-inferiortemporal - ctx-lh-parahippocampal |
| ctx-lh-middletemporal - ctx-lh-parahippocampal |
| ctx-lh-inferiortemporal - ctx-lh-superiortemporal |
| ctx-lh-parahippocampal - ctx-lh-superiortemporal |
| ctx-lh-lateralorbitofrontal - ctx-lh-temporalpole |
| ctx-lh-parahippocampal - ctx-lh-temporalpole |
| ctx-lh-parahippocampal - ctx-lh-transversetemporal |
| ctx-lh-fusiform - ctx-lh-insula |
| ctx-lh-inferiortemporal - ctx-lh-insula |
| ctx-lh-middletemporal - ctx-lh-insula |
| ctx-lh-parahippocampal - ctx-lh-insula |
| ctx-lh-superiortemporal - ctx-lh-insula |
| ctx-lh-temporalpole - ctx-lh-insula |
| ctx-lh-inferiortemporal - Left-Thalamus-Proper |
| ctx-lh-fusiform - Left-Putamen |
| ctx-lh-inferiortemporal - Left-Putamen |
| ctx-lh-parahippocampal - Left-Putamen |
| ctx-lh-entorhinal - Left-Pallidum |
| ctx-lh-inferiortemporal - Left-Pallidum |
| ctx-lh-superiortemporal - Left-Pallidum |

**Table S 4:** **Transsylvian sAH pre- vs postsurgical connectivity strength differences.** Edges showing a significant postsurgical (vs. presurgical) decrease in connectivity strength in patients who underwent transsylvian sAH (FWE-corr. p < 0.05). Abbreviations follow the standard FreeSurfer lookup table.

| Edges (node to node) | Edges (node to node) |
| --- | --- |
| ctx-lh-fusiform - ctx-lh-isthmuscingulate | ctx-lh-lingual - ctx-lh-insula |
| ctx-lh-fusiform - ctx-lh-lateralorbitofrontal | ctx-lh-middletemporal - ctx-lh-insula |
| ctx-lh-inferiortemporal - ctx-lh-lateralorbitofrontal | ctx-lh-parahippocampal - ctx-lh-insula |
| ctx-lh-entorhinal - ctx-lh-lingual | ctx-lh-parsorbitalis - ctx-lh-insula |
| ctx-lh-fusiform - ctx-lh-medialorbitofrontal | ctx-lh-superiortemporal - ctx-lh-insula |
| ctx-lh-inferiortemporal - ctx-lh-medialorbitofrontal | ctx-lh-temporalpole - ctx-lh-insula |
| ctx-lh-caudalanteriorcingulate - ctx-lh-middletemporal | ctx-lh-caudalmiddlefrontal - Left-Thalamus-Proper |
| ctx-lh-lateralorbitofrontal - ctx-lh-middletemporal | ctx-lh-fusiform - Left-Thalamus-Proper |
| ctx-lh-medialorbitofrontal - ctx-lh-middletemporal | ctx-lh-inferiortemporal - Left-Thalamus-Proper |
| ctx-lh-entorhinal - ctx-lh-parahippocampal | ctx-lh-rostralmiddlefrontal - Left-Thalamus-Proper |
| ctx-lh-inferiortemporal - ctx-lh-parsorbitalis | ctx-lh-superiortemporal - Left-Thalamus-Proper |
| ctx-lh-middletemporal - ctx-lh-parsorbitalis | ctx-lh-temporalpole - Left-Thalamus-Proper |
| ctx-lh-inferiortemporal - ctx-lh-parstriangularis | ctx-lh-transversetemporal - Left-Thalamus-Proper |
| ctx-lh-middletemporal - ctx-lh-parstriangularis | ctx-lh-fusiform - Left-Caudate |
| ctx-lh-fusiform - ctx-lh-rostralanteriorcingulate | ctx-lh-parsorbitalis - Left-Caudate |
| ctx-lh-entorhinal - ctx-lh-rostralmiddlefrontal | ctx-lh-superiortemporal - Left-Caudate |
| ctx-lh-fusiform - ctx-lh-rostralmiddlefrontal | ctx-lh-entorhinal - Left-Putamen |
| ctx-lh-inferiortemporal - ctx-lh-rostralmiddlefrontal | ctx-lh-fusiform - Left-Putamen |
| ctx-lh-fusiform - ctx-lh-superiorfrontal | ctx-lh-inferiortemporal - Left-Putamen |
| ctx-lh-entorhinal - ctx-lh-superiortemporal | ctx-lh-middletemporal - Left-Putamen |
| ctx-lh-fusiform - ctx-lh-superiortemporal | ctx-lh-parahippocampal - Left-Putamen |
| ctx-lh-inferiortemporal - ctx-lh-superiortemporal | ctx-lh-parsorbitalis - Left-Putamen |
| ctx-lh-lingual - ctx-lh-superiortemporal | ctx-lh-superiortemporal - Left-Putamen |
| ctx-lh-medialorbitofrontal - ctx-lh-superiortemporal | ctx-lh-temporalpole - Left-Putamen |
| ctx-lh-middletemporal - ctx-lh-superiortemporal | ctx-lh-entorhinal - Left-Pallidum |
| ctx-lh-parahippocampal - ctx-lh-superiortemporal | ctx-lh-fusiform - Left-Pallidum |
| ctx-lh-parsorbitalis - ctx-lh-superiortemporal | ctx-lh-inferiortemporal - Left-Pallidum |
| ctx-lh-middletemporal - ctx-lh-frontalpole | ctx-lh-lateraloccipital - Left-Pallidum |
| ctx-lh-entorhinal - ctx-lh-temporalpole | ctx-lh-middletemporal - Left-Pallidum |
| ctx-lh-lingual - ctx-lh-temporalpole | ctx-lh-superiortemporal - Left-Pallidum |
| ctx-lh-parahippocampal - ctx-lh-temporalpole | ctx-lh-temporalpole - Left-Pallidum |
| ctx-lh-parahippocampal - ctx-lh-transversetemporal | ctx-lh-transversetemporal - Left-Pallidum |
| ctx-lh-entorhinal - ctx-lh-insula | ctx-lh-inferiortemporal - Left-Accumbens-area |
| ctx-lh-fusiform - ctx-lh-insula | ctx-lh-middletemporal - ctx-rh-fusiform |
| ctx-lh-inferiortemporal - ctx-lh-insula | ctx-lh-fusiform - ctx-rh-superiortemporal |

| Model | TP | FP | TN | FN | Specificity | Sensitivity | PPV | NPV | Accuracy | F1 score | *p* |
| --- | --- | --- | --- | --- | --- | --- | --- | --- | --- | --- | --- |
| ANN meta-classifier + surgerical procedure | 20 | 1 | 7 | 0 | 85.71 | 100 | 95.24 | 100 | 96.43 | 0.98 | *0.02 |
|  |  |  |  |  |  |  |  |  |  |  |  |
|  |  |  |  |  |  |  |  |  |  |  |  |
|  |  |  |  |  |  |  |  |  |  |  |  |
|  |  |  |  |  |  |  |  |  |  |  |  |
|  |  |  |  |  |  |  |  |  |  |  |  |
|  |  |  |  |  |  |  |  |  |  |  |  |
|  |  |  |  |  |  |  |  |  |  |  |  |
|  |  |  |  |  |  |  |  |  |  |  |  |
|  |  |  |  |  |  |  |  |  |  |  |  |

**Table S 5: Performance of meta classifier with surgical procedure information.** (TP = true positives, FP = false positives, TN = true negatives, FN = false negatives, PPV = positive predictive value, NPV = negative predictive value)

| 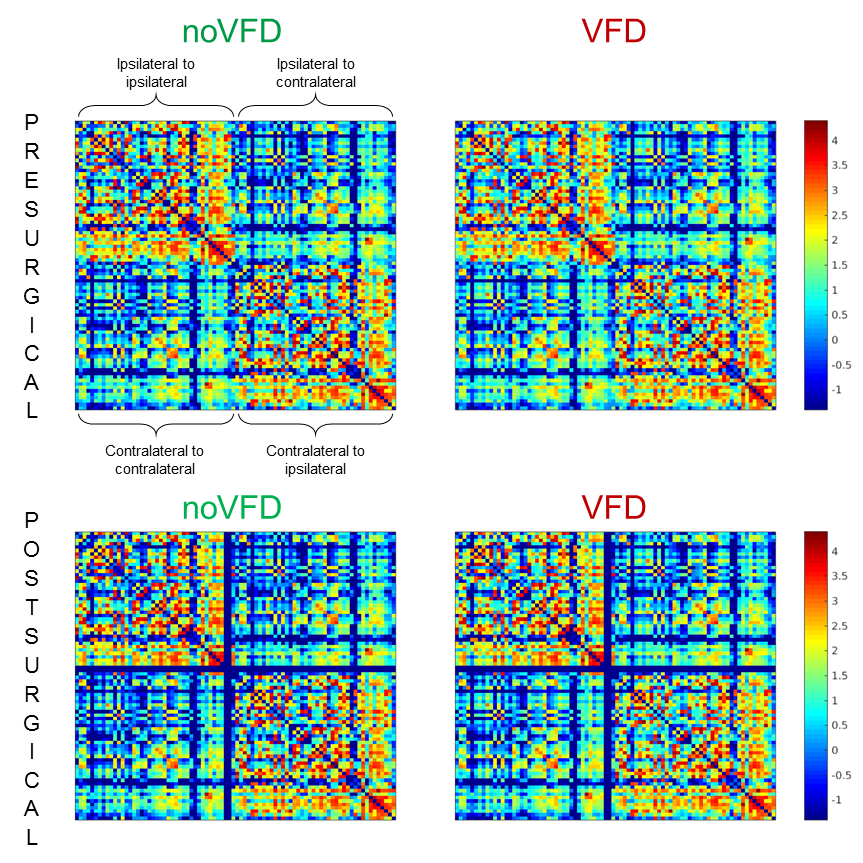 |
| --- |
| **Fig. S1:** **Mean connectivity matrices.** Connectivity matrices before and after selective amygdalohippocampectomy for patients with and without postsurgical VFD. Color intensities indicate the mean streamline count for the specific edges in the connectome on a logarithmic scale. |

| 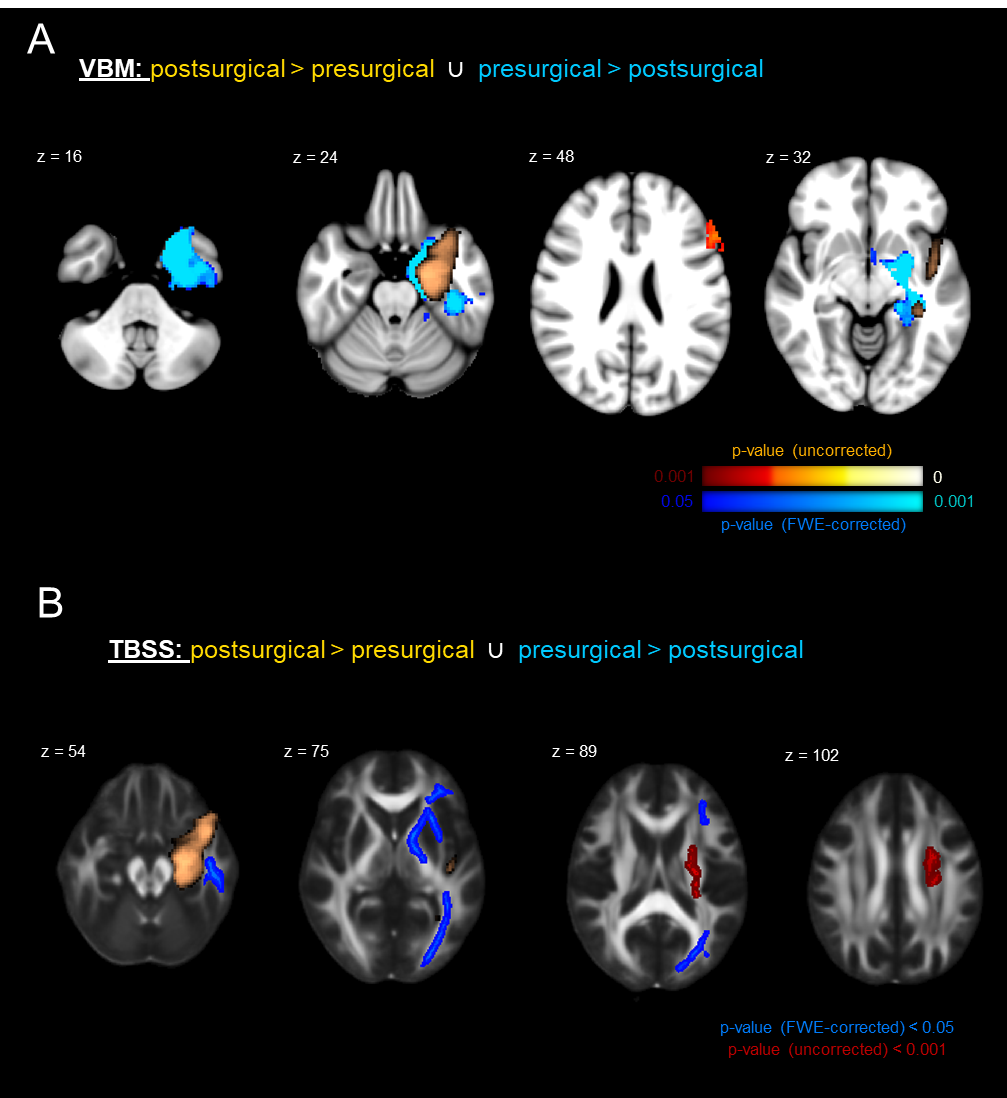 |
| --- |
| **Fig. S2:** **Voxelwise results for patients with a surgery-scan-interval < 12 months. A:** Gray matter volume comparison of pre- and postsurgical T1-weighted scans of the transsylvian patient subgroup. **B:** Comparison of fractional anisotropy in pre- and postsurgical FA maps of the transsylvian patient subgroup. |
